# Persistent carriage of MRSA in pig farmers

**DOI:** 10.1186/1753-6561-5-S6-P167

**Published:** 2011-06-29

**Authors:** BV Cleef, BV Benthem, R Teuwen, J Kluytmans

**Affiliations:** 1 Centre for Infectious Disease Control Netherlands, RIVM National Institute for Public Health and the Environment, Bilthoven, Netherlands; 2 Department of Medical Microbiology and Infection Prevention, VU University Medical Centre, Amsterdam, Netherlands; 3 Laboratory for Microbiology and Infection Control, Amphia Hospital, Breda, Netherlands

## Introduction / objectives

Livestock associated methicillin-resistant *Staphylococcus aureus* (MRSA) is a known and prevalent pathogen related to livestock farming. Little is known about the dynamics of carriage over time in pig farmers.

## Methods

In this prospective cohort study, pig farmers and their employees from 50 pig farms in The Netherlands were tested for MRSA presence in nose and throat at 5 time points in 8 months. Questionnaires were taken as well. Persistent carriers were defined as persons with 100% of nasal samples positive for MRSA. Multilevel multivariate regression analysis was performed in SAS. This is a preliminary analysis as the collection of data is still ongoing.

## Results

In total, 130 pig farmers and employees entered the study (101 males, 78%). Eighty of them (62%) were MRSA nasal carriers at the start of the study and 49 (38%) were persistent carriers. Presence of MRSA in throat samples at the start of the study (OR=6.1, 95% CI 1.6-23.6), giving birth assistance to sows (OR=8.1, 95% CI 1.9-35.3), and the presence of goats on the farm (OR=6.0, 95% CI 1.0-35.7) were significantly associated with persistent carriage. Amount of working hours per week did improve the model fit, but was not a significant factor (OR=1.0, p=0.18).

## Conclusion

The vast majority of persons working with pigs on a daily base is carrier of MRSA and almost 40% are persistent carriers. Throat carriage and specific tasks or farm characteristics were determinants of persistent carriage.

## Disclosure of interest

None declared.

